# Dissection flap fenestration can reduce re-apposition force of the false lumen in type-B aortic dissection: a computational and bench study

**DOI:** 10.3389/fbioe.2024.1326190

**Published:** 2024-03-28

**Authors:** Aashish Ahuja, Xiaomei Guo, Jillian N. Noblet, Joshua F. Krieger, Blayne Roeder, Stéphan Haulon, Sean Chambers, Ghassan Kassab

**Affiliations:** ^1^ California Medical Innovations Institute, San Diego, CA, United States; ^2^ Cook Medical, Bloomington, IN, United States; ^3^ Chirurgie Vasculaire—Centre de l’Aorte, Hôpital Marie Lannelongue, Université Paris Saclay, Paris, France

**Keywords:** aortic dissection, slit fenestration therapy, simulation, FEA, stent graft, acute dissection, type-B dissection, bench validation

## Abstract

Thoracic endovascular aortic repair (TEVAR) has been widely adopted as a standard for treating complicated acute and high-risk uncomplicated Stanford Type-B aortic dissections. The treatment redirects the blood flow towards the true lumen by covering the proximal dissection tear which promotes sealing of the false lumen. Despite advances in TEVAR, over 30% of Type-B dissection patients require additional interventions. This is primarily due to the presence of a persistent patent false lumen post-TEVAR that could potentially enlarge over time. We propose a novel technique, called slit fenestration pattern creation, which reduces the forces for re-apposition of the dissection flap (i.e., increase the compliance of the flap). We compute the optimal slit fenestration design using a virtual design of experiment (DOE) and demonstrate its effectiveness in reducing the re-apposition forces through computational simulations and benchtop experiments using porcine aortas. The findings suggest this potential therapy can drastically reduce the radial loading required to re-appose a dissected flap against the aortic wall to ensure reconstitution of the aortic wall (remodeling).

## 1 Introduction

Thoracic endovascular aortic repair (TEVAR) has become the gold standard for treating complicated, acute, and high-risk uncomplicated Type B aortic dissection (TBAD). Aortic dissection (AD) is classified as Stanford type-B if it originates distal to the left subclavian artery and does not involve the ascending aorta. In type-B dissection, there is a separation and propagation within the media layer where blood enters the aortic wall to create a false channel, known as the false lumen (FL), in addition to the normal endothelialized channel referred to as the true lumen (TL). The layer of the aorta dissected from its wall is referred to as the dissection flap. The adoption of TEVAR treatment has significantly reduced the mortality of complicated TBAD as compared to open surgical repair. Although short-term results are promising, the long-term outcomes still need to be more optimal, calling into question the ability of current endovascular solutions to provide complete aortic remodeling. According to the International Registry of Acute Aortic Dissection (IRAD), at 5 years post-TEVAR, aortic growth or new aneurysm formation is present in 60% of patients, and freedom from reintervention stands at only 40% (Fattori, et al., 2013). Several factors have been linked to long-term aortic growth after type-B dissection, namely, the persistence of FL perfusion from distal re-entry tears, absence of either multiple entries or distal re-entry (Trimarchi, et al., 2014). Complete re-apposition of the flap and sealing of all distal re-entry tears can improve long-term outcomes of TEVAR for TBAD.

Here, we propose a solution for the dissection flap by creating a slit fenestration pattern that minimizes the re-apposition force of the flap or increases the compliance of the flap. This is a method of creating a well-defined pattern of slits in the dissection flap and then performing TEVAR using a stent/graft device to re-appose the intimal flap to its outer wall. This potential solution advances prior techniques (Hartnell and Gates, 2005) of creating a hole in the distal part of the dissection flap (also termed “fenestration”) using balloon angioplasty (Panneton, et al., 2000; Clair, 2002; Wuest, et al., 2011; Vendrell, et al., 2015), dividing the flap longitudinally (termed as “septectomy”) using a wire snare (Beregi, et al., 2000; Barshes, et al., 2015; Berguer, et al., 2015) or disrupting the intimal flap by using an inflated balloon inside a stent/graft (also termed as “knickerbocker technique” (Kölbel, et al., 2014) or “stent-assisted balloon-intimal disruption” (Hofferberth, et al., 2014)). These techniques offer quick restoration of branch vessel perfusion but have failed to prevent continual aortic aneurysmic growth after endovascular treatment. The outcomes are not surprising since the re-apposition and sealing of the FL are critical for the strength of the aorta to prevent aortic dilation over time, and a single-hole fenestration is likely insufficient to increase the compliance of the flap sufficiently for complete re-apposition with a stent or a graft. Also, due to FL dilation after TBAD, septectomy produces an area with a reduced wall thickness, vulnerable to aneurysmal degeneration over time. Therefore, a more mechanically-designed solution is needed to promote the re-apposition of the intimal flap and stabilization of the aortic wall.

This study aims to introduce a novel slit fenestration pattern therapy and demonstrates that this potential therapy can reduce the radial loads required to re-appose the dissected flap against the FL wall (i.e., increase the compliance of the flap) after TEVAR. Virtual design of experiments (DOE) using a full factorial simulation design was conducted to choose an optimal slit fenestration pattern. Benchtop experiments utilize the chosen optimal design of slit fenestration pattern to validate the observations from simulations.

## 2 Materials and methods

### 2.1 Finite element modeling of the dissected aorta

#### 2.1.1 Model geometry and pre-Processing steps

**FIGURE 1:**
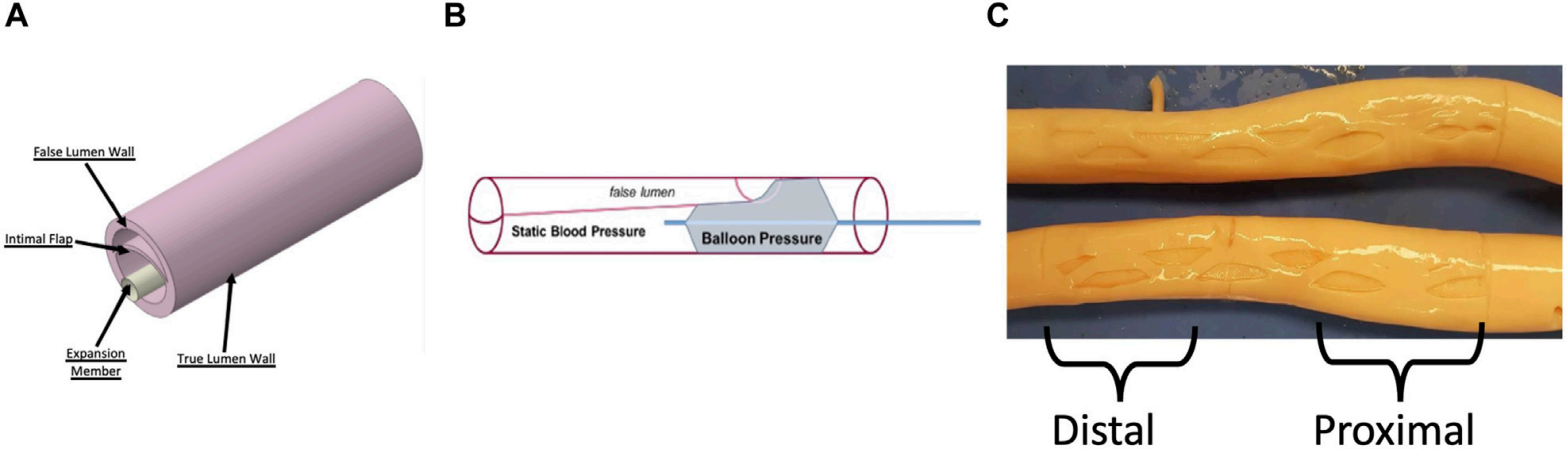
**(A)** CAD geometry of a dissected aorta and the rigid expansion member used to re-appose the flap against the FL wall. Adapted from (Ahuja et al., 2018a) **(B)** Schematic of the benchtop setup used to record re-apposition pressure. A dissected porcine aorta was pressurized at static pressure, and a balloon was placed within the TL and inflated to re-appose the flap against the FL wall **(C)** Inverted and Dissected Aortas with the optimal slit fenestration pattern. The proximal and distal sections of the aorta are mentioned, which are also areas of interest for bench tests and simulations.

An idealized model of a dissected aorta was prepared in Solidworks (v2017, Dassault Systemes). and simulated in Abaqus (v6.13.5, Dassault Systemes) using the Abaqus/Standard non-linear solver. The 3D CAD geometry for a dissected porcine aorta is shown in Figure 1A, and the different tissue regions are labeled. The CAD consists of three layers: TL wall, FL wall, and dissection flap. For each dissected aortic region, we use the anisotropic Holzapfel-Gasser-Ogden (HGO) hyperelastic constitutive material model (Gasser et al., 2006). The HGO models the mechanical contribution of distributed collagen fibers in each layer of the aorta during deformation. The parameters for the different layers of the dissected aorta corresponding to proximal and distal regions of the aorta are presented in Table 1 and were adopted from (Ahuja et al., 2018a).

**TABLE 1:** Parameter Estimation for TL wall, Proximal flap and FL wall, and Distal flap and FL wall.

Region	Thickness (mm)	C10 (Pa)	k1 (Pa)	k2	α (degrees)	κ
TL wall	1.76	78,219	201,440	1.52	87.1	0.2
Proximal flap	0.58	92,963	230,290	13.90	87.1	0.33
Proximal FL wall	1.3	53,456	952,380	4.94	7.5	0.3
Distal flap	0.4	103,140	61,969	4.1	62.4	0.1
Distal FL wall	1.24	72,996	20,894	9.01	66.5	0

The inner walls of the aorta were subjected to an internal pressure of 50–140 mmHg. To model a nearly rigid expansion member within the dissected aorta’s true lumen, a long cylindrical body was used in simulations. Firstly, the inner walls of the aorta were pre-stressed to match the static pressurization applied during bench testing (as described in §2.2) by applying an internal pressure of between 50 and 140 mmHg. In the next step, while keeping the aortic pressure constant, the nearly rigid member was expanded using a displacement boundary condition to re-appose the intimal flap, which is similar to the inflation of a non-compliant balloon. During both simulation steps, the end faces of the dissected aorta vessel were constrained from moving along the axial direction. The circumferential length of the intimal flap was adjusted to be within 50%–60% of the aortic circumferential length (Ahuja et al., 2018b).

All the components of the model were meshed using reduced-order hybrid linear hexahedral elements (C3D8RH), except the expansion member, which was meshed using linear shell elements (S4). The intimal flap underwent bending due to contact force being applied by the expansion member. The hybrid solid elements in this simulation represent tissue materials, which are assumed to be incompressible and thus volume-conserving. The mesh elements undergo distortion but no dilatation. It is recommended to use reduced order with linear solid elements for problems where bending is the primary mode of deformation, such as the ones presented here. To avoid issues with contact interactions, the thicknesses across the different tissue regions (FL wall, TL wall, and intimal flap) were meshed with at least two rows of C3D8RH elements. Three contact interactions were established between 1) Expansion member and intimal flap 2) Expansion member and TL wall 3) Intimal flap and FL wall.

The linear shell elements (S4) are a good choice for modeling the cylindrical member, which expands axisymmetrically and has an isotropic material response. By using shell elements, the computational performance can be improved since there is no need to compute the stresses across the thickness of the thin cylinder.

A sensitivity analysis on mesh was also performed as part of the study which was introduced in (Ahuja et al., 2018a). A global element size of 0.3 mm was used for both the dissected aorta and the expansion member in all the considered cases. The radial pressure results at 100 mmHg were consistent within a 2.5% margin of error when compared to a refined mesh. The sensitivity of the mesh was tested by reassessing the distal flap re-apposition, and the results are presented here. The dissected aorta model, with a global element size of 0.3 mm, had 69,126 hexahedral elements. The radial pressure only decreased by 1.7% when compared to a model with a global element size of 0.2 mm (233,655 hexahedral elements). However, opting for the finer mesh size increased computational time by an average of 200% (from 0.63 h to 1.9 h) using a San Diego Supercomputer Center’s (SDSC) workstation with 24 cores. (Towns, et al., 2014).

#### 2.1.2 Design of experiment to search for an optimal slit fenestration pattern

A virtual design of experiments (DOE) was set up to choose an optimal slit fenestration pattern. The mechanical response of arterial layers such as intima, media, and adventitia stiffen at higher strains, owing to the stretching of collagen fibers (Gasser et al., 2006). This imposes higher radial pressures on the modified intimal flap and the healthy TL wall during the re-apposition process. Additional stretching of the intimal flap and TL wall results in increased strain energy, which can potentially cause long-term aortic growth. Our hypothesis states that the slit fenestration pattern can relieve the radial stresses generated in the intimal flap during the re-apposition process. The DOE enables us to determine an optimal slit fenestration pattern from multiple candidates for testing on *ex-vivo* tissue and future *in-vivo* studies.

Several independent process variables were considered in the DOE, which dictated the geometry of the slits produced on the intimal flap. The design search space consisted of the following process variables to produce different patterns:

1) Length of the slits relative to flap section.
2) Number of columns of vertical slits in a pattern (i.e. 1, 2 or 3 vertical slits per column).
3) Location of slits (X- and Y- coordinates) *w.r.t* centerline.
4) Number of slits arranged horizontally across each row (i.e. 1, 2 or 3 horizontal rows).
5) Vertical offset between rows of slits (for creating asymmetric patterns).

Twenty-eight simulations for the symmetric patterns and sixty-six simulations for the asymmetric patterns were executed to search for the optimal slit fenestration pattern that satisfies the objective of this full factorial DOE design. Supplementary Appendix A details all the different simulated patterns (a total of 94 slit patterns) to select the optimal slit fenestration pattern. The slit geometries modeled were constrained to patterns containing straight cuts through the thickness of the flap and parallel to the dissected aorta axis. This constraint was applied to make it simple to reproduce the same pattern on *ex-vivo* tissue.

A simulation was executed to represent the re-apposition process of the modified intimal flap against the FL wall for a pressurized aorta at 100 mmHg for every slit fenestration pattern design. Small fillets were introduced to avoid issues around convergence and singularity at the ends of the slits. The objective was to select an optimal slit fenestration pattern that generates the lowest average circumferential stress across the flap surface after re-apposition.

Two cases of simulation were run to validate the goal of this study. The first was the baseline (or control) with no fenestration, and the second included the optimal slit fenestration pattern on the intimal flap. To investigate the effects of applying internal static aortic pressures between 50–140 mmHg, several sub-cases of flap re-apposition were simulated for both cases.

#### 2.1.3 Post-Processing simulations

The contact pressure output (under field output label CPRESS) applied to the expansion member was computed from Abaqus for all static aortic pressures between 50–140 mmHg. An averaged contact pressure over the surface of the expansion member was calculated using a Python script. The radial pressures obtained for the baseline and slit fenestrated cases were compared to the re-apposition pressures measured during benchtop experiments.

### 2.2 Bench experiments for Re-apposition of intimal flap against FL wall

Using the benchtop model described in (Ahuja et al., 2018b), dissection was created in three samples of porcine aorta (see Supplementary Appendix B for geometrical specifications of the dissected aortas).

A series of experiments were conducted on dissected but non-fenestrated aortas (baseline) to quantify the loading required for the re-apposition of the flap. To demonstrate re-apposition, the CODA balloon was introduced into the proximal-mid TL (∼6–8 cm from LSA) and inflated with water under ultrasound imaging guidance (Philips, iE33) until the flap contacted the FL wall, as shown in Figure 1B. Pressure measurements were recorded using the sensors attached to it, and then the balloon was moved to the distal (∼10–14 cm from LSA) region of the TL, where it was inflated again, and new measurements were recorded. During the experiment, the entire balloon was contained within the dissection. The aorta was then pressurized to static pressures between 50–140 mmHg in increments of 10 mmHg.

The aortas were then inverted inside out, and the desired slit fenestration pattern was created on the flap using a surgical scalpel, resulting in aortas shown in Figure 1C. The aortas were re-inverted and remounted on the benchtop model fixture. Sample #2 was damaged during the creation of slit fenestration, and thus only baseline values were reported for this sample.

The setup described in Figure 1B was also used to investigate the decrease in radial loading required for re-apposing the intimal flap with a slit-fenestrated pattern against the FL wall. The experiments involving the re-apposition of the aorta, with or without a slit fenestration pattern, were recorded at a range of static aortic pressures. The balloon pressure minus the aortic pressure estimated the pressure required for re-apposition.

**FIGURE 2:**
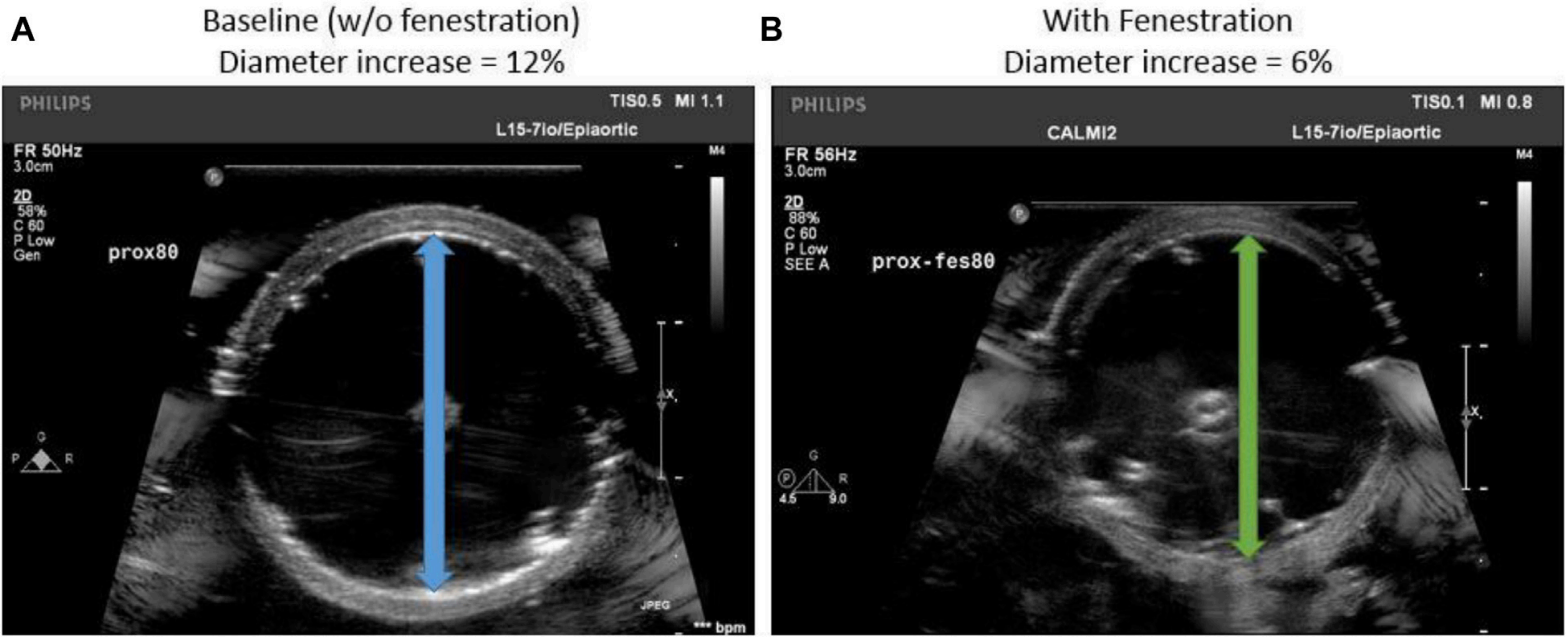
**(A)** Transverse ultrasound image of the baseline aorta suspended in the benchtop setup **(B)** An aorta flap fenestrated with optimal slit fenestration pattern is re-apposed using a balloon.

Ultrasound images of the *ex-vivo* aorta was also captured for both the baseline and fenestration cases as shown in Figure 2. The ultrasound imaging allowed tracking of geometry (e.g., diameter) and stiffness (diameter-pressure relation) of the aorta in real-time during testing of various fenestration patterns.

### 2.3 Statistics

Our study aims to investigate the effect of two factors on the re-apposition pressure observed during bench tests. These two factors are the presence or absence of slit-fenestrated flaps and the application of aortic pressure ranging from 50–140 mmHg. We used five aorta specimens, three of which were non-slit fenestrated, and the remaining two had slit-fenestrated flaps. Since our sample size was small and the data was not evenly distributed, we used a non-parametric two-way ANOVA (Conover and Iman, 1981) to analyze the data ranks. This type of statistical analysis is useful when the normality assumption is unmet. We also applied similar analyses on data ranks for the re-apposition pressures observed during bench tests and computational simulation results.

We conducted independent linear regressions on datasets from both proximal and distal aortas (bench tests and computational). All datasets had a good fit, and the slopes for each dataset were recorded.

### 2.4 Selection of optimal slit fenestration pattern

**FIGURE 3:**
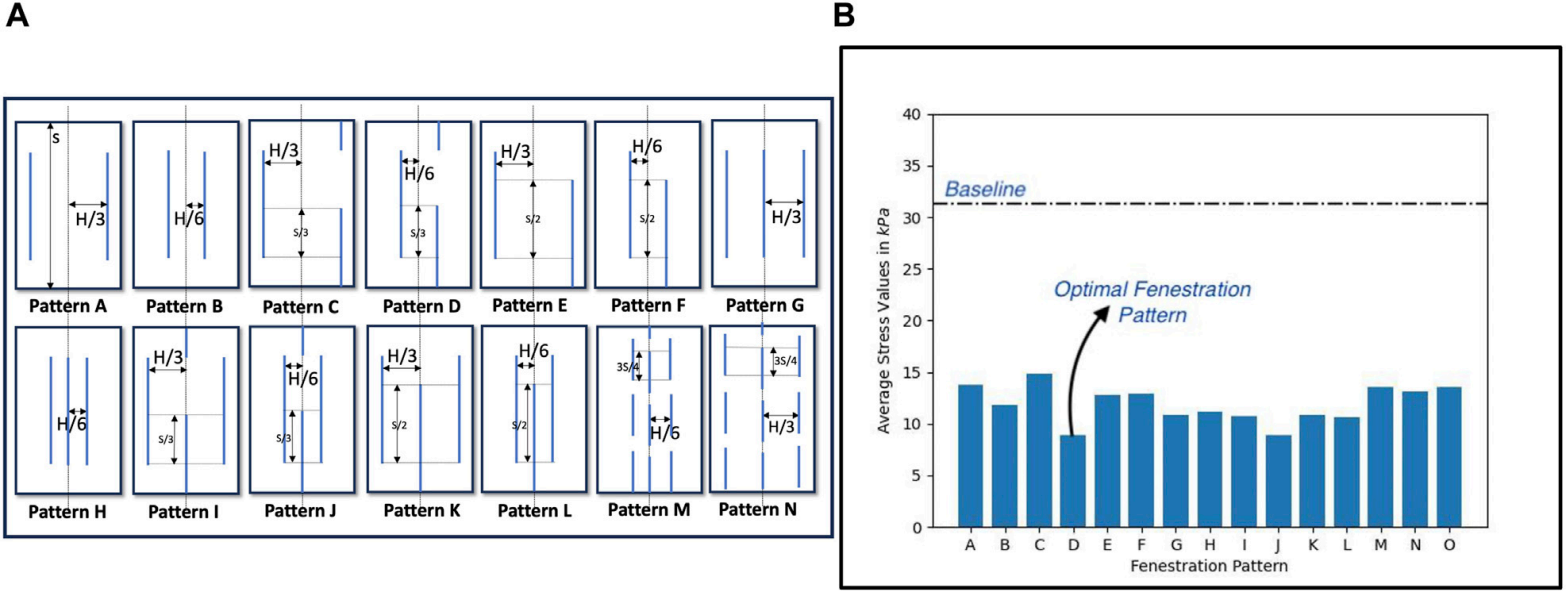
**(A)** Different slit fenestration patterns and the **(B)** average circumferential stresses generated during re-apposition simulations.

The average circumferential stresses for the slit fenestration patterns presented in Figure 3A were plotted in Figure 3B. Supplementary Appendix A provides a schematic representation of all the considered pattern designs. Patterns D and J produced the lowest average circumferential stress values. Since Pattern D had two rows instead of three rows for Pattern J, Pattern D was selected as it was easier to recreate for benchtop experiments. Overall, it was observed that flaps with slit fenestration patterns experienced lower average circumferential stresses than the baseline, un-fenestrated aortas.

**FIGURE 4:**
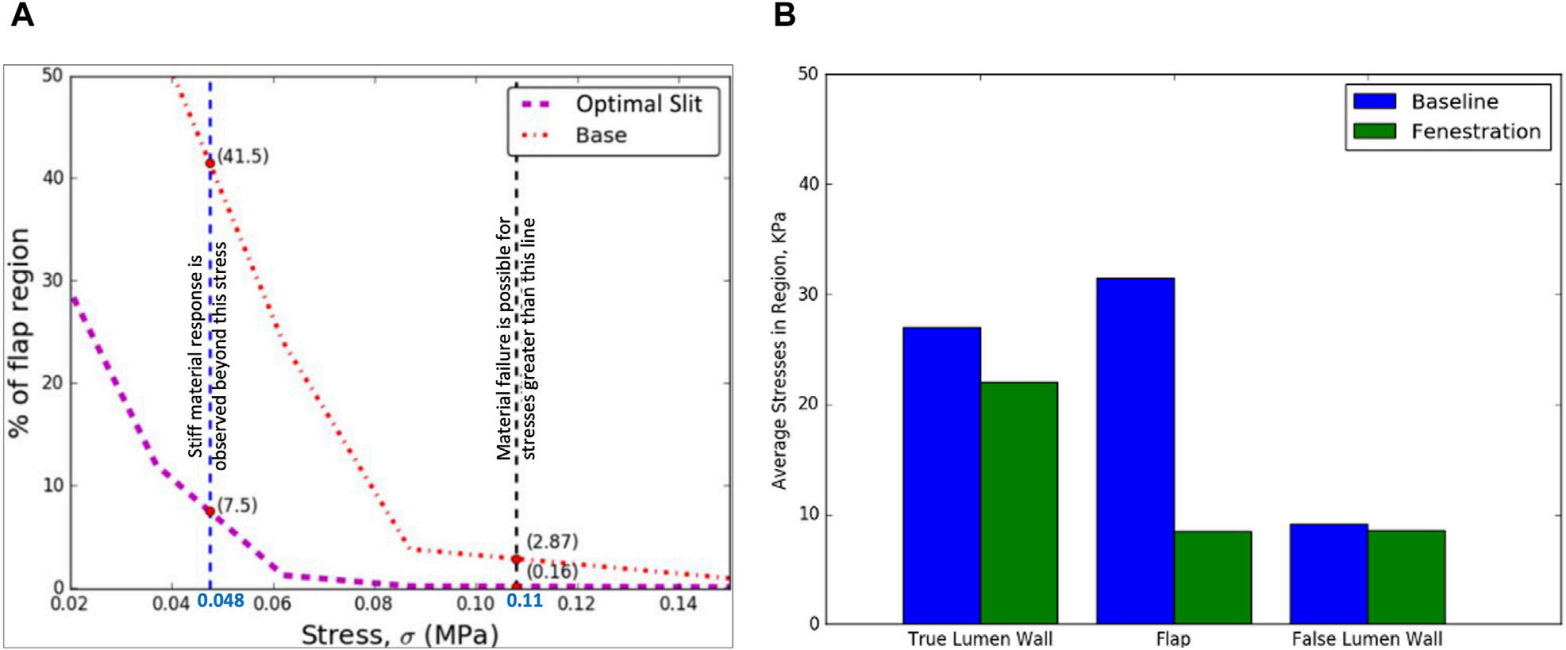
**(A)** The optimal slit fenestration pattern lowers the peak circumferential stresses generated in the flap during re-apposition **(B)** Average circumferential stress reduction in TL wall, flap, and FL wall during the re-apposition process for the non-fenestrated baseline case and in the presence of slit fenestration Pattern D.

Peak circumferential stress calculations for optimal Pattern D were computed, depicted in Figure 4A. It can be observed that compared to the baseline (non-fenestrated) case, a fenestration flap with an optimal slit fenestration pattern has 34% lesser surface area that contains stresses exceeding the values at which the tissue specimen starts to stiffen considerably. Further, virtually no slit-fenestrated intimal flap area contains stresses above the failure stress value as measured from experimental testing, potentially leading to tearing or fracture. The reductions in average and peak circumferential stresses support that the inclusion of the slit fenestration pattern D increases the compliance of the flap.

The increased compliance of the intimal flap with slit fenestration Pattern D facilitated the complete re-apposition of the flap against the FL wall. Moreover, an additional advantage of creating a slit fenestration pattern is reduced loading imposed on the TL wall during re-apposition. Figure 4B shows a 73% decrease in average circumferential stresses in the flap and a 19% decrease in the TL wall stresses due to Pattern D.

### 2.5 Ultrasound measurements of aorta after Re-apposition procedure

**FIGURE 5:**
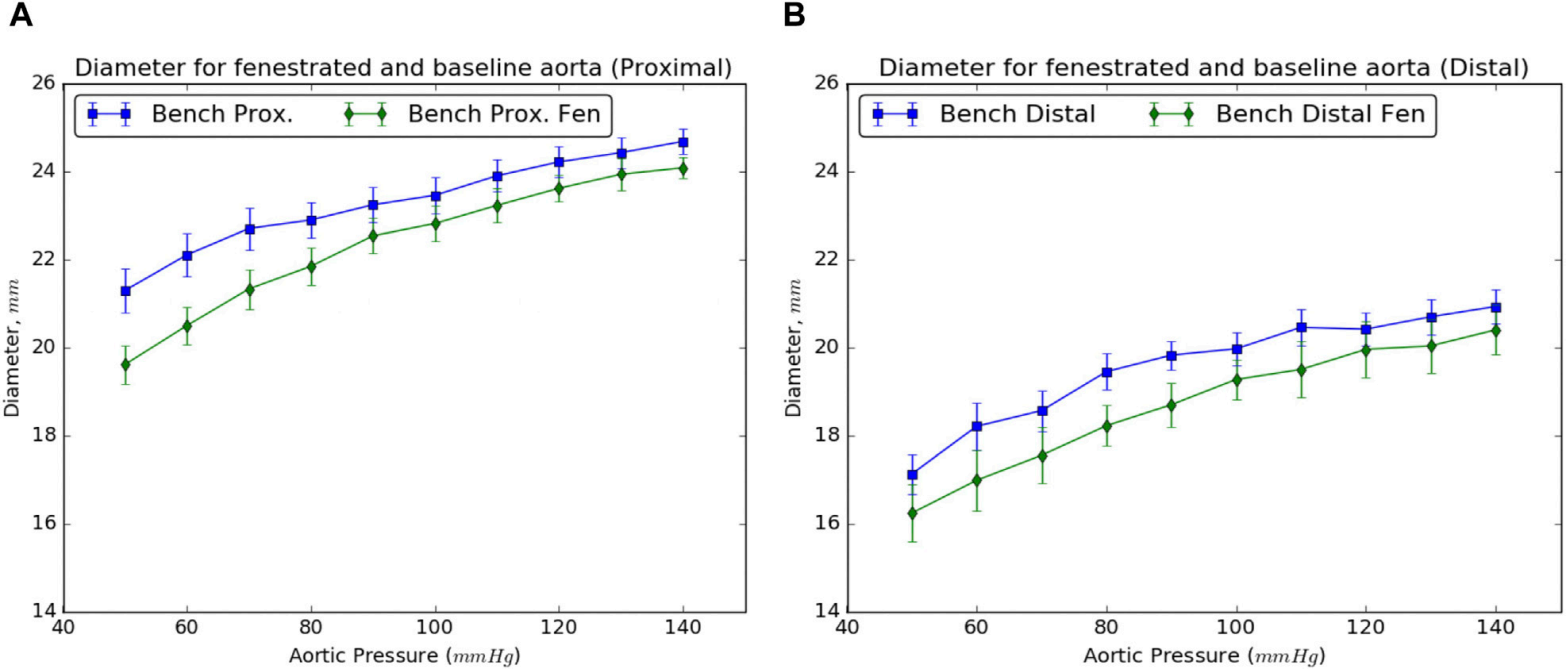
Resulting diameters of the **(A)** proximal and **(B)** distal sections of the dissected aorta on complete re-apposition of the intimal flap in the presence and absence of slit fenestration.

The values for the diameter of the aorta after re-apposition were measured from ultrasound images captured during bench testing. The images were captured at the start of the experiment (when the balloon was completely deflated) and when the flap was in complete contact with the FL wall. It should be noted that the diameter of the distal part of the aorta always has a smaller value than the proximal or mid region of the aorta for all static aortic pressures imposed during bench testing. From Figures 2, 5, it was found that in the presence of slit fenestration Pattern D, there was a reduction in the radial pressures, resulting in a smaller aortic diameter compared to the baseline case. Thus, a smaller balloon or endograft would be required to re-appose the flap against the FL wall for both proximal and distal cases. The difference between the aortic diameters after re-apposition for the baseline case and in the case with pattern D decreases as the aortic pressures increase. The reason is that higher aortic pressures dictate the final diameters of the vessel, which is independent of the presence of a fenestration pattern.

### 2.6 Benchtop experiment results

The setup described in the schematic in Figure 1B was used to calculate the radial pressures required to completely re-appose the intimal flap against the FL wall in the presence or absence of a slit fenestration pattern over the range of static aortic pressures between 50–140 mmHg. The pressure inside the aorta is directly transmitted through the CODA balloon (CODA-2-9.0-35-100-32, Cook Medical, Indiana), and thus, there is aortic pressure inside the balloon—even when the balloon is minimally inflated. Thus, the pressure required for re-apposition is the delta between the balloon and aortic pressures. This delta pressure may inform an expansion device such as a stent/graft, which does not need to overcome aortic blood pressure to achieve re-apposition.

**FIGURE 6:**
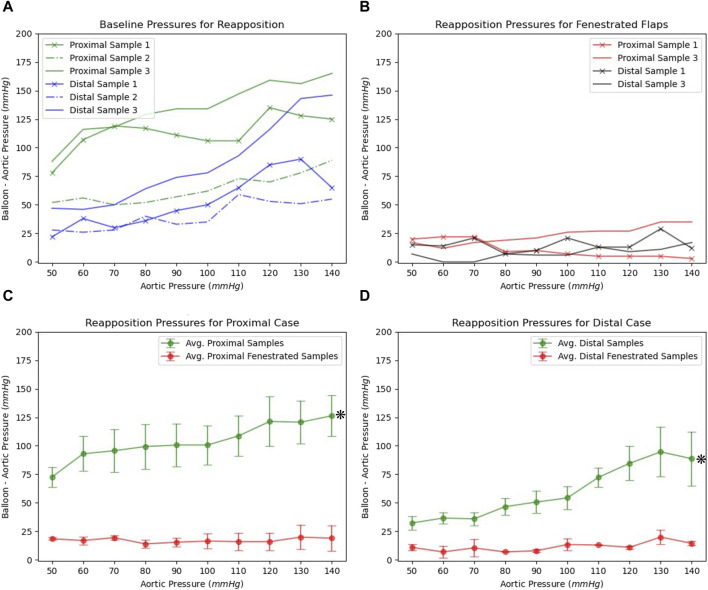
**(A)** The re-apposition pressures required for baseline dissected aorta over the range of static aortic pressures **(B)** Re-apposition pressures for slit fenestrated aortas under different static aortic pressures. Average and standard errors for re-apposition pressures observed for baseline and slit-fenestrated flaps tested in **(C)** proximal and **(D)** distal regions.

For the baseline case, a steady increase in re-apposition pressure with increasing aortic pressure was observed from bench tests (shown in Figure 6A). This is due to an increase in static aortic pressure, resulting in an overall increase in the diameter of the vessel, putting additional tension on the flap and thereby increasing the pressure required for re-apposition. Differences in overall pressure required to re-appose between the proximal and distal regions reflect the reduced aortic diameter and flap thickness in the distal segment.

In the presence of slit fenestration pattern D, the re-apposition pressure for the aorta shown in Figure 1C is markedly reduced over the range of static aortic pressures tested. This is also consistent with findings from computational simulations. Furthermore, the re-apposition pressure is independent of the aortic pressures applied internally to the FL and TL walls, as observed in Figure 6B. This implies that including a slit fenestration pattern alters the deformation mechanism of the flap from tensile circumferential strain to bending loads on the tissue between the slits. Statistical significance (*p* < 0.05) was observed between baseline and slit-fenestrated flaps in both proximal and distal regions in the two-way ANOVA as shown in Figures 6C,D. However, there was no statistical significance (*p* > 0.05) observed when comparing responses at different aortic pressures.

**FIGURE 7:**
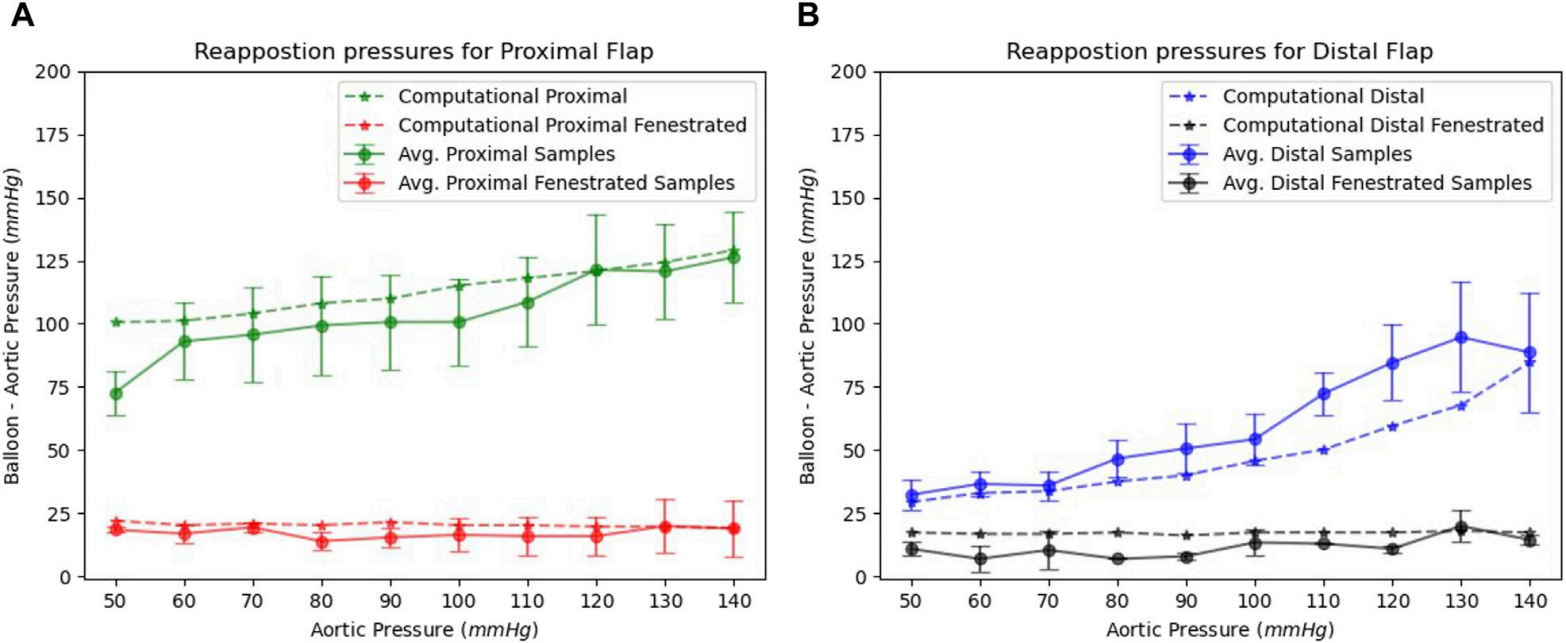
Benchtop and computational re-apposition pressures are required to re-appose **(A)** proximal and **(B)** distal flap sections against the FL wall. The re-apposition pressures are significantly reduced in the presence of slit fenestration.

Finally, a comparison between experimental bench studies and *in silico* computational studies were made as shown in Figure 7. The comparison was made for both the proximal and distal flaps with and without slit fenestration. Statistical analysis of both proximal and distal sections showed no statistical significance (*p* > 0.05), which was visually confirmed in Figure 7.

We created four groups based on slopes of re-apposition pressure datasets from proximal and distal baseline and slit-fenestrated flaps. All the groups passed normality tests with *p* > 0.05. We calculated the 95% confidence intervals (CI) for each group as follows: proximal baseline {0.28, 0.65}, distal baseline {0.35, 1.05}, proximal slit-fenestrated flap {−0.27, 0.26}, and distal slit-fenestrated flap {−0.02, 0.14}. The 95% CI for baseline and slit-fenestrated flaps do not overlap. Moreover, the CI for slit-fenestrated proximal and distal flaps contain zero slopes, suggesting that the applied static aortic pressure weakly affects or does not affect the re-apposition pressures for flaps with the slit-fenestrated pattern.

## 3 Discussion

The most important finding of the present study is that fenestration of the flap using relatively simple patterns can substantially reduce the re-apposition force or pressure of the flap. Both the experimental and computational studies confirmed our hypothesis that optimally designed fenestration patterns can increase the compliance of the flap to re-appose the flap and reconstitute the aortic wall.

The FL wall, dissected flap, and TL wall were characterized using a Holzapfel-Gasser-Ogden (HGO) hyperelastic constitutive material model (Gasser et al., 2006). Since the intimal flap undergoes significant bending stresses due to the distension of the expansion member, different material properties were applied to characterize the proximal-mid and distal flap regions. The material definitions and parameters for the material models of different tissue constituents were adopted from (Ahuja et al., 2018a).

The results from bench experiments indicate an increase in the diameter of the dissected aorta with aortic pressure. Specifically, the diameter of the dissected aorta is affected by two reasons: 1) Increase in the aortic pressure results in an overall increase in the diameter of the vessel, and 2) Increase in aortic diameter puts additional tension on the flap, which increases the net balloon pressure required to re-appose the flap. This trend was observed for all aortic pressures considered in Figure 5, requiring a larger balloon diameter to completely re-appose the flap against the wall.

It should be noted that the dissections created in aortic samples comprised of largely intimal and medial layers. Hence, the exact pressure values may not be directly applicable to all TBAD cases. Larger distension and thicker flap from increased TBAD depth may require higher re-apposition pressure. The study has been focused on the creation of slit fenestration patterns on the flap regions of TBAD that are affected by TEVAR. This will enable sealing the FL from a patent flow with lower reapposition forces. Any remaining dissected aortic flap regions that are not part of the TEVAR, however, would not undergo slit fenestration pattern creation. Moreover, the impact of introducing slit fenestration to descending aortic branches has not been examined, which is currently beyond the scope of this study.

In the baseline cases, there was a notable difference between the pressures needed for re-apposing the proximal and distal flaps against the FL wall. A significant decrease in the pressure required for re-apposition was observed, however, after making slit fenestration Pattern D on the aorta samples. This phenomenon was demonstrated in the plots in Figure 6B. The plot data and the range of revealed that aortas with slit fenestration had a re-apposition pressure mostly unaffected or weakly affected by the aortic pressure. This finding emphasizes that slit fenestration alters the deformation mechanism from flap strain to tissue bending between the slits. The final re-apposition pressures were similar for the proximal and distal sections of the slit-fenestrated flap.

Finally, it was mentioned previously that the re-apposition occurs at a smaller aortic diameter in the presence of slit fenestration (Figures 2, 5), presumably associated with lower stresses on the flap and TL wall during re-apposition. This reduced stress due to slit fenestration may eventually reduce the risk of aortic dilation and aneurysm formation post-TEVAR. However, further experiments and simulations are required to assess the validity of this statement.

To create a slit fenestration pattern clinically, a tissue-cutting system can be used. This system may include a conductive probe and an expandable structure made of a conducting material like a balloon. The conductive probe and the conducting material can be positioned on opposite sides of the aortic dissection flap. By applying electrical power to a desired wattage, an appropriate slit pattern on the flap can be created. A patent design application for such kind of a system can be found in (Roeder, et al., 2018).

## 4 Limitations

The bench test data for baseline and slit fenestrated cases were collected from *ex-vivo* samples of porcine aorta at room temperature. During the creation of the slit fenestration pattern, sample #2 was damaged, resulting in a smaller sample size that was inadequate for statistical analysis. Also, it is important to note that the ambient conditions during the bench test differ from those during a re-apposition procedure performed *in-vivo* at body temperature. The simulations also utilized material properties for the different constituents of a dissected aorta, such as FL and TL walls and intimal flap. The testing for these materials was also conducted at room temperature, which might affect the data on their mechanical response.

In our experiments and simulations, we quantified the radial loading needed to re-appose the flap in the presence of static aortic pressure. In the human body, the dissected flap of the aorta undergoes dynamic movements due to variations in aortic pressure during the cardiac cycle. This will affect the movement of the flap and the TL and FL walls. Due to cardiac cycles, radial loading required to seal FL and the efforts to place stent/graft might differ from simulations and benchtop tests. The process of creating a slit fenestration technique may affect the diseased FL wall, which could result in complications like aortic dilation or aneurysms. Therefore, it is imperative to conduct future studies that assess vessel durability and mid-term complications after creating an optimally designed fenestration pattern. The effectiveness of slit fenestration therapy may be affected by the presence of multiple re-entries and TBAD extending to descending aortic branches, requiring further evaluation.

The computational simulations used geometrically idealized geometry to dissect the aorta for DOE and develop re-apposition models. The utilization of a reconstructed model from imaging such as echocardiography or CT scanning would have provided granular insights into the efficacy of slit fenestration therapy in sealing the FL. A high-resolution 3D model can be reconstructed by acquiring cross-sectional images for a dissected aorta with a thickness of around 1 mm/slice (Ohbuchi and Fuchs, 1991). The dissection for bench testing was created manually by initiating a tear and then advancing a surgical blade, resulting in a straight flap. TBAD inside the body are instantaneous and spiral within the aorta (Armstrong, et al., 1996) as it progresses from the thoracic to the abdominal aorta. Further research through bench studies and computational models will be necessary to confirm the effectiveness of slit fenestration therapy in assisting TEVAR for dissection anatomies with rotated flaps.

## 5 Conclusion

A design of experiments was used to find the best pattern for slit fenestration. This pattern should produce the least amount of circumferential stress in the dissected flap when it is re-attached to the FL wall. Adding the slit fenestration in the flap also helped reduce bending stresses and stress on other parts of the aorta during balloon inflation. Computational simulations and benchtop experiments showed that the pressure required to re-appose a slit fenestrated flap remained constant regardless of internal aortic blood pressure or re-apposition location.

Additional pre-clinical studies and simulations are required to verify if TEVAR with the addition of slit fenestration has further benefits, such as: 1) Restoration of aortic wall mechanical properties and blood flow hemodynamics; 2) Reduction of the risk of aortic dilation and aneurysm formation; and 3) Decrease in re-intervention rates among patients. These potential benefits remain to be demonstrated in pre-clinical and clinical studies.

## Data Availability

The original contributions presented in the study are included in the article/Supplementary Material, further inquiries can be directed to the corresponding author.
