# Supplementary material for: Dissection flap fenestration can reduce re-apposition force of the false lumen in type-B aortic dissection: a computational and bench study
Source: Front Bioeng Biotechnol. 2024 Mar 28;12:1326190. doi: 10.3389/fbioe.2024.1326190 (PMC11007646; doi:10.3389/fbioe.2024.1326190)
Supplement: Supplementary file 1 [file DataSheet1.docx]

**Appendix A**

1. Summary

In bench and computational testing, ‘Slit Fenestration Pattern Creation therapy’ showed promise by relieving the bending stresses generated in the Intimal flap during re-apposition. This appendix presents a methodical procedure called ‘Design of Experiments (DOE)’ to optimize the slit fenestration pattern that generates the least bending stresses during re-apposition.

1. Scope

The scope of these tests is to conduct computational modeling on optimizing the slit fenestration pattern and show strong evidence for using this pattern in advancing therapy for treating aortic dissection.

1. References

The simulation models were developed and executed using the commercial CAE software Abaqus/Standard v2017. The models were prepared using the steps provided in ‘Ahuja et al (2018) Validated Computational Model to Compute Re-apposition Pressures for Treating Type-B Aortic Dissections. Front. Physiol. 9:513’

1. Objective

The objective is to find the optimal slit fenestration pattern among several designs based on the criterion that it generates the lowest average circumferential stress value.

1. Method

The following slit fenestration patterns were considered:

Class of designs with total overlap between columns of slits:

*Sample #1: Slits arranged along the centerline*

| Length of slits (%flap length, S) | Number of slits along each column | | |
| --- | --- | --- | --- |
|  | 1 | 2 | 3 |
|  | 33% | 33% | N/A |
|  | 50% | N/A | N/A |
|  | 66% | N/A | N/A |

Note: The pattern designs that are marked as ‘N/A’ are not physically possible.


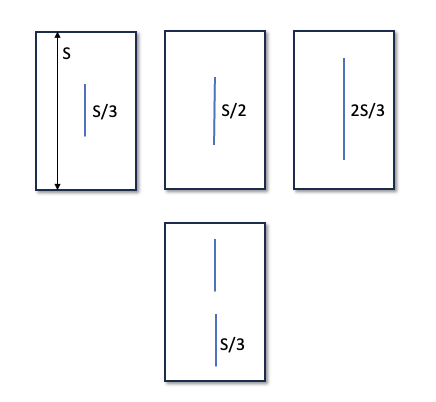


Figure: Slits arranged along the centerline

*Sample #2: Slits arranged along two and three columns*

| Length of slits (%flap length, S) | Number of slits along each column | | |
| --- | --- | --- | --- |
|  | 1 | 2 | 3 |
|  | 33% | 33% | N/A |
|  | 50% | N/A | N/A |
|  | 66% | N/A | N/A |

An additional parameter was used to define how far apart columns are located relative to the centerline. For three columns, one of the columns are located along the centerline. Three options were explored:

- 1. Columns of slits are positioned 33% of the half-flap (H/6) width from the centerline.
  2. Columns of slits are positioned 50% of the half-flap (H/4) width from the centerline.
  3. Columns of slits are positioned 66% of the half-flap (H/3) width from the centerline.


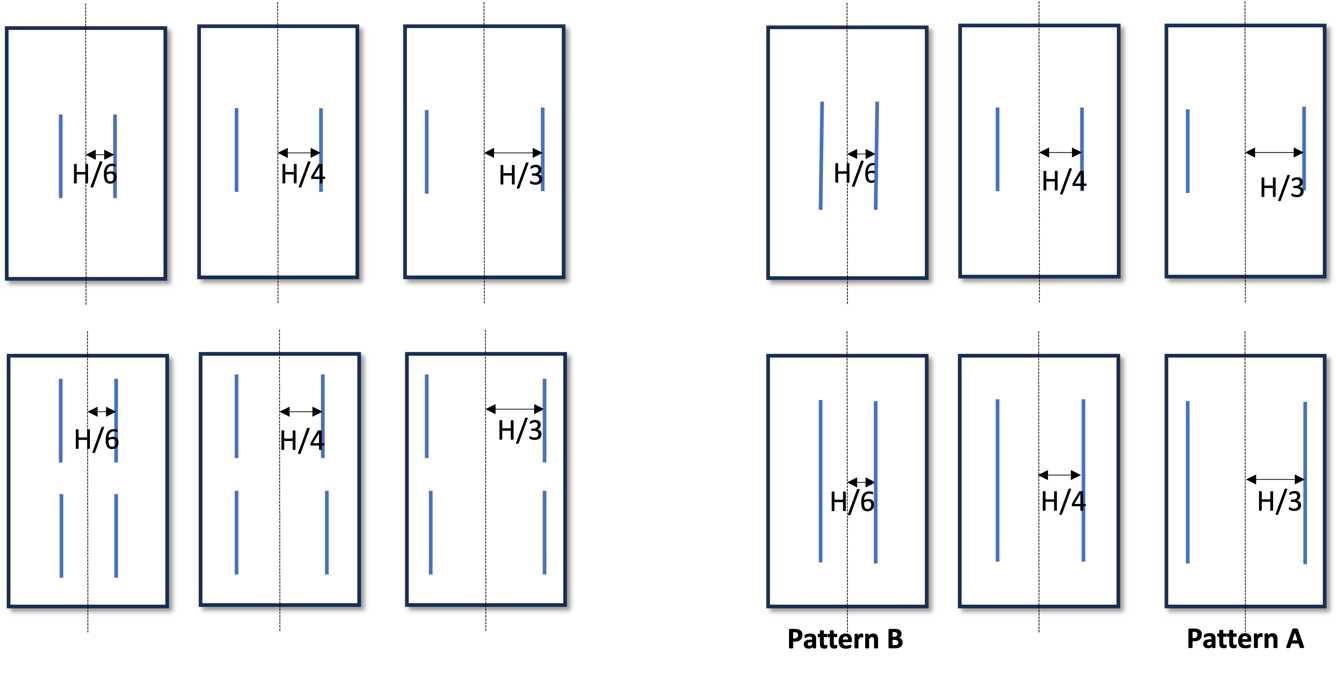


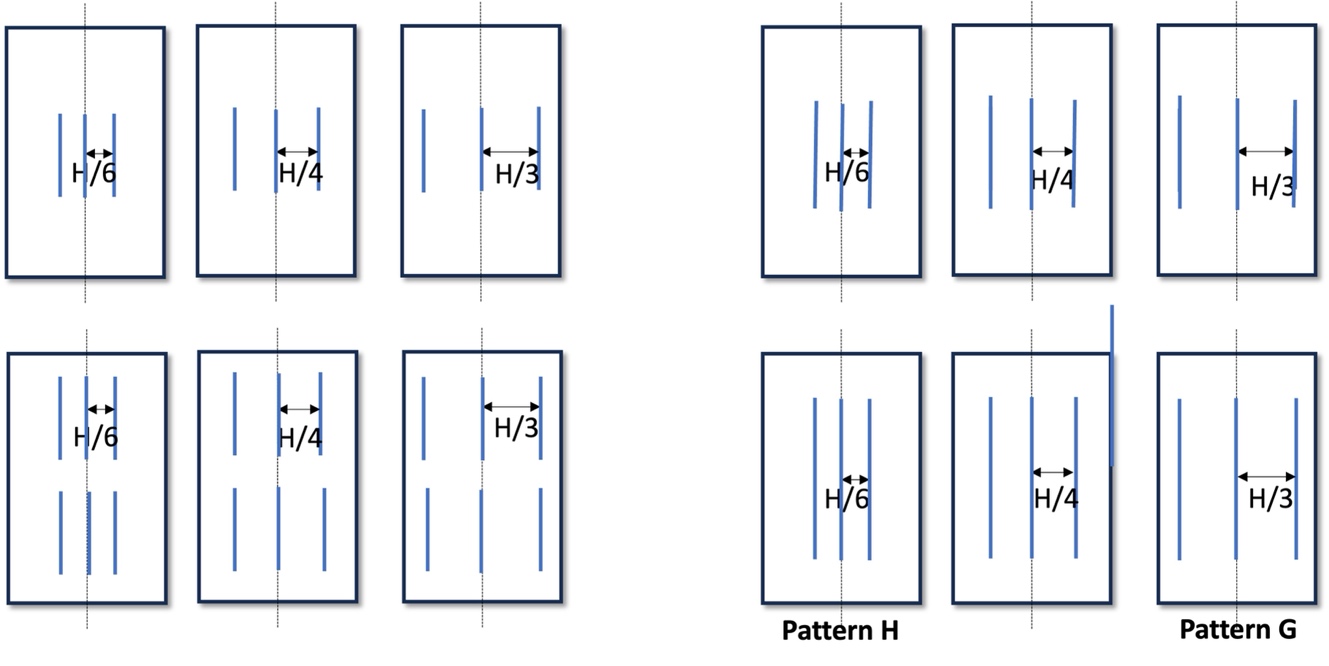


Figure: Slits arranged along two and three columns creating a total overlap between slits

Class of designs with less than 100% overlap between columns of slits

*Sample #1: Slits arranged along two and three columns*

| Length of slits (%flap length, S) | Number of slits along each column | | |
| --- | --- | --- | --- |
|  | 1 | 2 | 3 |
|  | 33% | 33% | 25% |
|  | 50% | N/A | N/A |
|  | 66% | N/A | N/A |

In addition to the placement of columns with respect to the centerline, a parameter to define the overlap conditions of 25%, 50%, and 75% between slits were also applied. Two constraint conditions were applied between slits:

- For a slit that covers 66% of flap length, a minimum overlap of 50% is attained.
- In the case where two slits are present along a column, the minimum overlap is 50%.
- In the case where three slits are present along a column, the minimum overlap used is 75%.


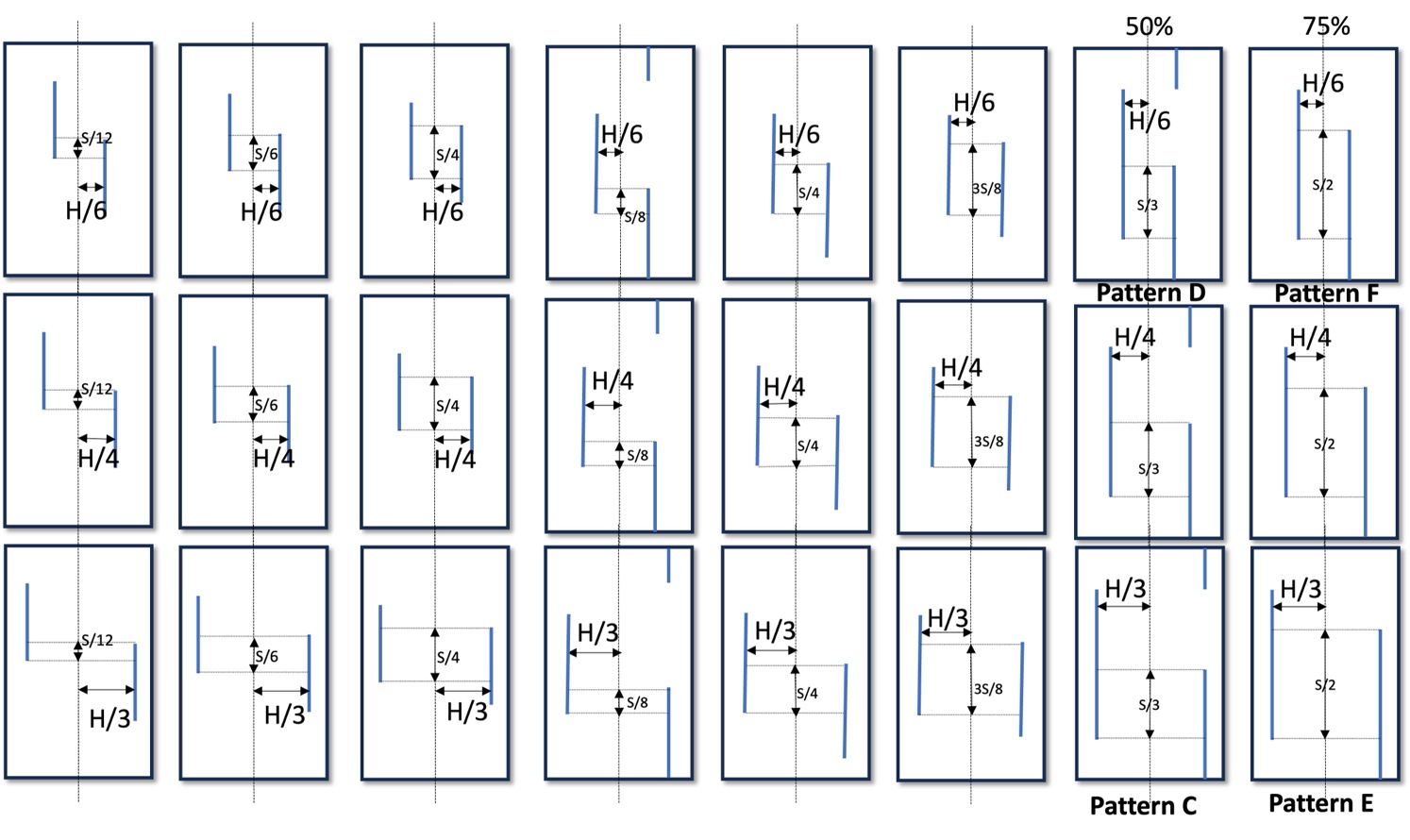


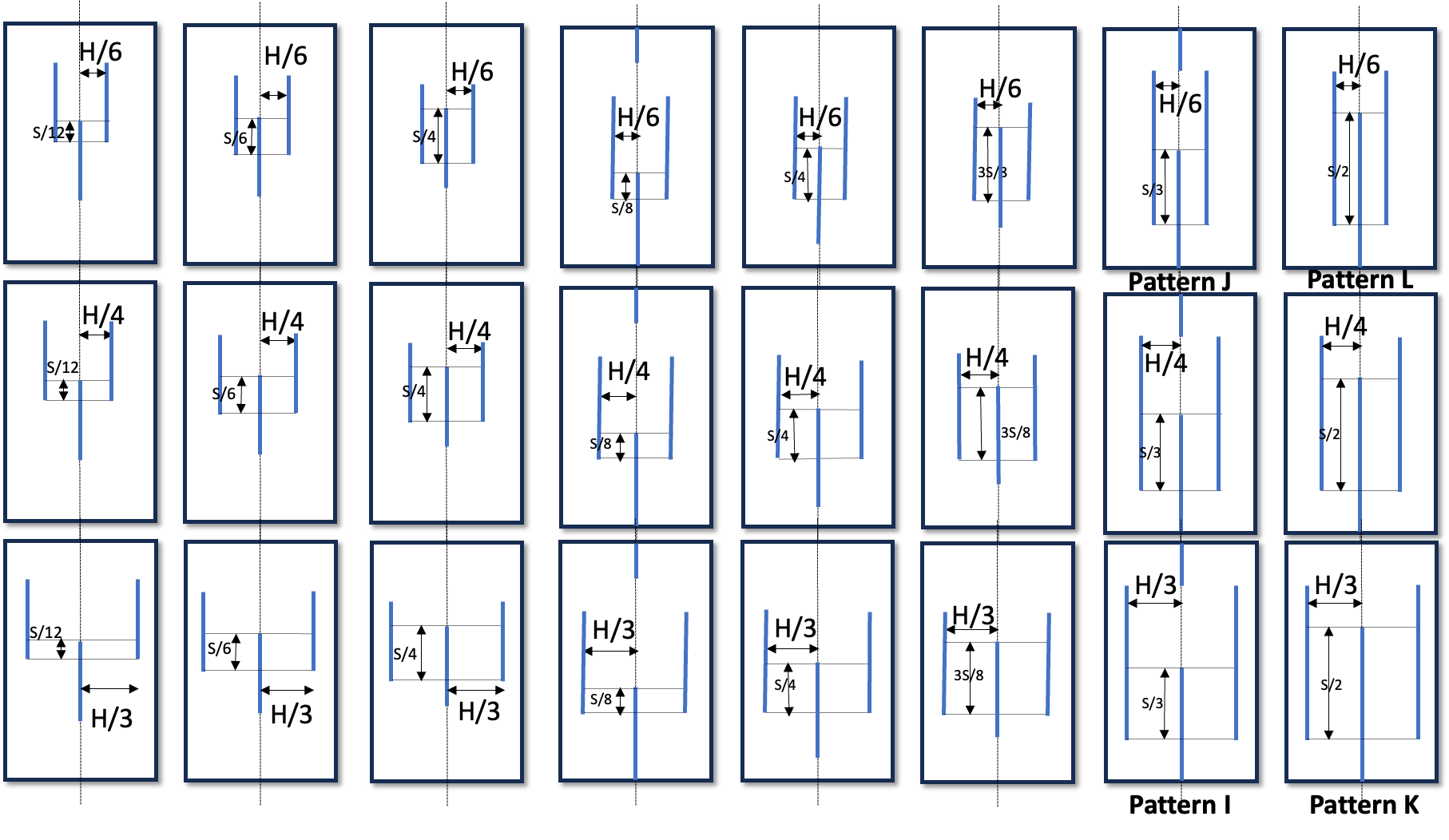


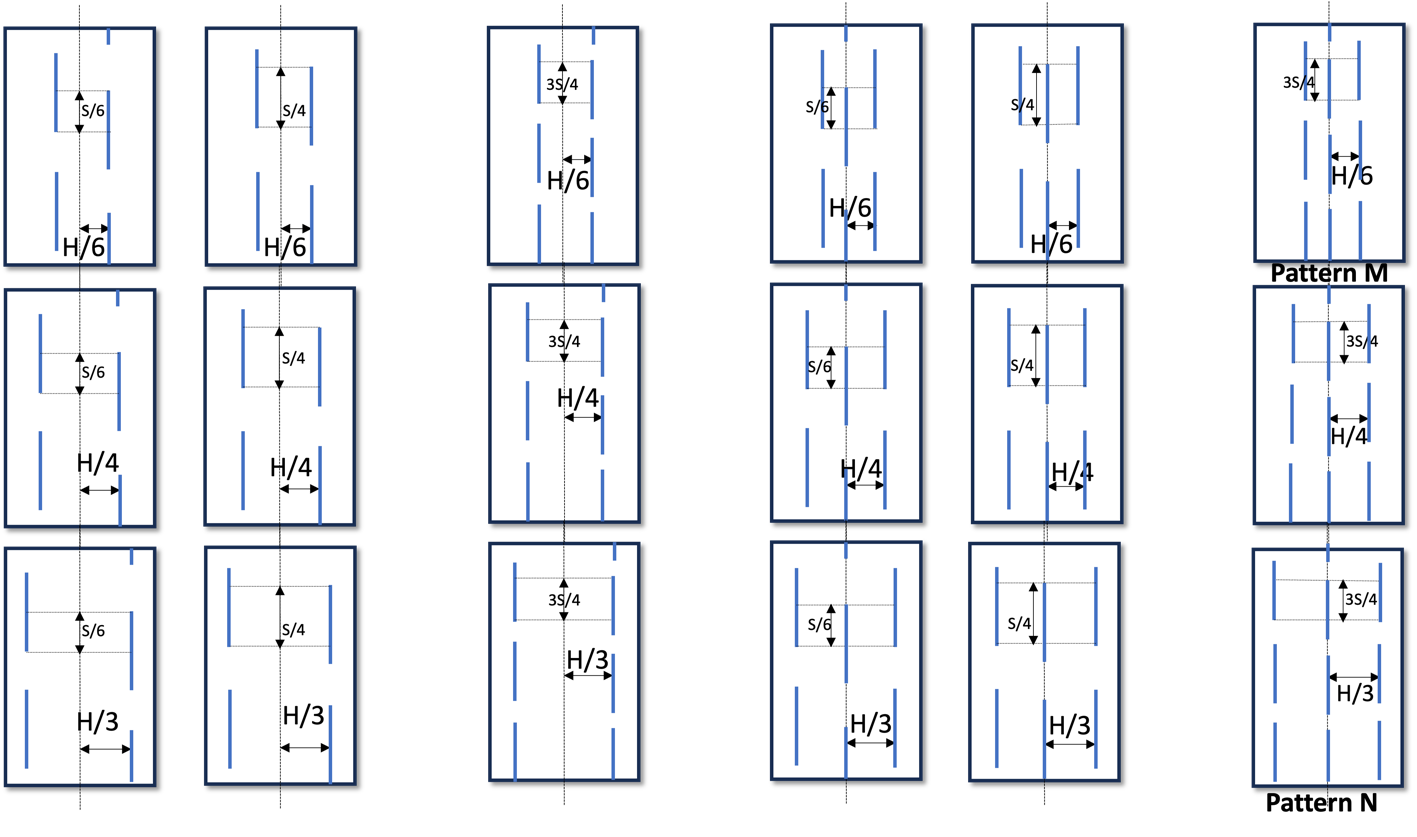


Figure: Slits arrangement such that there is less than 100% overlap between columns of slits

**Appendix B**

1. **Summary**

To evaluate the effects of including slit fenestration in reducing the pressure required to re-appose the flap to the outer wall, using benchtop experiments

1. **Scope**

Computational simulations have shown that slit fenestration significantly reduces the pressure required to re-appose the flap with the outer wall. This testing is designed to repeat this preliminary testing with a Cook CODA balloon (CODA-2-10.0-35-140-46).

1. **Sample Description**

Table 1 – Test Sample Description

| Sample ID | Proximal Wall Thickness (mm) | Proximal Flap Thickness (mm) | Distal Wall Thickness (mm) | Distal Flap Thickness (mm) | Avg. Flap Percent Wall Thickness (%) |
| --- | --- | --- | --- | --- | --- |
| 1 | 2.04 | 0.45 | 1.71 | 0.37 | 22% |
| 2 | 2.29 | 0.53 | 1.55 | 0.29 | 21% |
| 3 | 2.07 | 0.64 | 1.44 | 0.48 | 32% |

1. **Sample Preparation & History**

Samples were prepared by removing all connective tissue and ligating all branches using 00 suture. All samples were prepared and tested within 72 hours. Samples were stored at ~2°C until tested. Samples were not used for any testing prior to the assessments stated below.

1. **Test Protocol**

The following steps were performed:

1. Create an initial incision
2. Create dissection
   1. Use forceps to create a dissection approximately 10 cm long
3. Create Re-entry
   1. Create a re-entry of approximately 25% the distal circumference
   2. Reinvert sample
4. Mount sample on static pressure fixture
5. Advance the CODA balloon into the proximal section of the dissection. Ensure the balloon working length is completely contained within the dissected length.
6. Pressurize the mounted sample to each of the following values
7. For each of the above sample pressure values:
   1. Inflate balloon until flap is apposed against outer wall
   2. Confirm flap re-apposition via ultrasound imaging
   3. Measure and record balloon pressure
8. Repeat steps 6-8 in the distal section of the sample.
9. Remove test sample from fixture and invert.
10. Create desired slit fenestration pattern in flap.
11. Reinvert and remount on fixture.
12. Repeat steps 6-9.
